# Sleep Disturbance Among Nurses in Al-Dakhiliyah Governorate, Oman

**DOI:** 10.7759/cureus.98704

**Published:** 2025-12-08

**Authors:** Abdullah Al Nabhani, Mohammed Al Harrasi, AlSalt Al Tubi, Ali Z Al Busaidi, Khalil Al Kharusi, Taleb Al Busaidi, Iman Al Furqani

**Affiliations:** 1 Psychiatry and Behavioral Sciences, Nizwa Hospital, Nizwa, OMN; 2 Internal Medicine, Nizwa Hospital, Nizwa, OMN; 3 Emergency Medicine, Nizwa Hospital, Nizwa, OMN; 4 Critical Care, Nizwa Hospital, Nizwa, OMN; 5 Statistics, Bahla Hospital, Bahla, OMN; 6 Nursing, Nizwa Hospital, Nizwa, OMN

**Keywords:** fatigue management, insomnia, insomnia severity index (isi), nurses, oman, shift work, sleep health

## Abstract

Background: Nurses are at high risk of insomnia due to demanding schedules and psychosocial stressors. We investigated the prevalence, severity, and predictors of insomnia among nurses working in Al-Dakhiliyah Governorate, Oman.

Methods: A cross-sectional survey of registered nurses (n=271) used a demographic/work-related questionnaire and the 7-item Insomnia Severity Index (ISI). Descriptive statistics, t-tests/ANOVA, and multiple linear regression were used (α=0.05). An online link was sent to the nurses, directing them to a Google Forms survey. The form included an informed consent section, a brief explanation of the study, and a statement emphasizing the anonymity of their responses.

Results: Clinically significant insomnia (ISI≥15) affected 24.0% of nurses, while 33.6% had subthreshold insomnia. Mean ISI was 9.46±6.30 (subthreshold range). In adjusted models (R²=0.253; adjusted R²=0.152), two independent predictors remained significant: nationality (non-Omani lower vs Omani; B=−2.7, p=0.028) and presence of medical illness (B=4.32, p<0.001). Night shifts were highly prevalent (84.1%) but not independently associated with ISI. The most severe ISI item was dissatisfaction with recent sleep.

Conclusions: Insomnia burden among nurses is substantial. Addressing co-morbid medical conditions and culturally informed stressors among Omani nurses should be prioritized. Multimodal programs (sleep hygiene, Cognitive Behavioral Therapy for Insomnia (CBT-I), fatigue management, optimized scheduling) are recommended.

## Introduction

Insomnia-difficulty initiating, maintaining sleep or dissatisfaction with sleep despite adequate opportunity-impairs cognition, vigilance and well-being, and increases risk of errors and burnout in healthcare providers [1,2,3,4]. The Sultanate of Oman’s health system relies heavily on nurses across hospitals and primary care [1,2]. Nurses in Al-Dakhiliyah Governorate face unique occupational and sociocultural demands that may predispose them to sleep disturbance [3]. Recent evidence highlights that insomnia and fatigue among healthcare workers are driven not only by long working hours but also by systemic factors such as workload intensity and irregular shifts, which substantially impair alertness and well-being [5]. Effective fatigue-management strategies in healthcare settings, like meditation, have been shown to reduce sleep-related impairment, emphasizing the need for organizational policies that support adequate rest and recovery [1,5]. In parallel, behavioral interventions such as mindfulness meditation have demonstrated significant improvements in sleep quality, suggesting that non-pharmacological approaches can play a critical role in addressing insomnia among healthcare staff [6]. Randomized trial evidence shows that mindfulness-based practices reduce insomnia symptoms by lowering physiological arousal and enhancing stress regulation, offering a promising method for sleep enhancement in high-stress professions such as nursing [5,6]. This study, therefore, aims to determine the prevalence and severity of insomnia among nurses working in Al-Dakhiliyah Governorate, to examine the demographic and occupational factors associated with insomnia among nurses and to propose evidence-based strategies to reduce insomnia and improve sleep quality among nurses.

## Materials and methods

Study design and time period

A descriptive, quantitative, cross-sectional study design was employed to determine the prevalence and associated factors of insomnia among registered nurses. The study was conducted in the Al Dakhiliyah Governorate of the Sultanate of Oman, encompassing government hospitals and primary health centres within the region. The study was conducted during October and November 2025.

Setting, sampling and participants

The study population consisted of all registered nurses currently employed in government hospitals and primary health centres across the Al-Dakhiliyah Governorate, Oman. The estimated total number of eligible nurses was approximately 1,800. A stratified random sampling technique was adopted to ensure proportional representation of nurses from different healthcare settings (hospitals and primary health centres). The sample size was calculated using Cochran’s formula with a finite population correction (FPC), based on an expected prevalence, a 95% confidence level, and a 6% margin of error. The minimum required sample size was 233. To account for a potential 10% non-response rate, the final target sample size was increased to 258 participants. Ultimately, 271 nurses participated. Nursing supervisors, students, interns, and administrative staff were excluded.

Data collection and measures

Data were collected between October and November 2025 using a structured self-administered Google Form (Appendix A). The survey consisted of sociodemographic and occupational variables (age, gender, nationality, marital status, number of children, years of experience, workplace level, night shift duties, medical illness, and use of sleep medication), and the validated seven-item Insomnia Severity Index (ISI) [7]. Each item is scored 0-4, yielding a total score range 0-28, with established cut-points (0-7 no insomnia, 8-14 subthreshold, 15-21 moderate, 22-28 severe).

Statistical analysis

Data analysis was conducted in SPSS Statistics (IBM Corp., 2019 IBM SPSS Statistics for Windows Version 26.0), and the results were displayed in the format of charts and tables. Descriptive statistics were used to summarize the data. To assess the normality of the ISI score, skewness and kurtosis values were examined, along with graphical methods such as histograms and Q-Q plots. Given the normal distribution of this variable, t-tests and ANOVA were utilized to explore the relationship between ISI score and background characteristics. Subsequently, variables were incorporated into a multiple linear regression analysis to identify predictors of ISI scores. A significance level of p<0.05 was established for all statistical tests.

Ethical considerations

Institutional approval for conducting the research was obtained from the Research and Ethical Review and Approval Committee, Al-Dakhliya Governorate, Ministry of Health, Sultanate of Oman, with approval number MоH/CSR/25/3099. Participation was voluntary, without incentives. Submission of the online survey constituted implied consent, and no identifying personal data was collected.

## Results

Sociodemographic characteristics of participants

A total of 271 nurses from Al-Dakhaliah Governorate participated in the study. Table 1 summarizes their demographic profile. The table shows that the majority of participants were Non-Omani (N=172, 63.5%), while 36.5% (N=99) were Omani. 

**Table 1:** Sociodemographic characteristics of participants (n=271). Local H.C: local health centre, HTN: hypertension, DM: diabetes mellitus.

Nurses’ characteristics	Category	Frequency	Percentage
Nationality	Omani	99	36.5%
	Non-Omani	172	63.5%
Workplace	Local HC	26	9.6%
	Polyclinic	11	4.1%
	Hospital	234	86.3%
Gender	Male	36	13.3%
	Female	235	86.7%
How many years worked as a nurse?	1-4	28	10.3%
	5-9	52	19.2%
	14-10	77	28.4%
	15-20	86	31.7%
	21-30	28	10.3%
Age group	20-29	27	10.0%
	30-39	163	60.1%
	40-49	76	28.0%
	50-59	5	1.8%
	Above 60	0	0.0%
How many years worked as a nurse at the current department?	1-5	155	57.2%
	6-10	43	15.9%
	11-15	45	16.6%
	16-20	16	5.9%
	21-25	9	3.3%
	26-30	3	1.1%
Marital status	Married	250	92.3%
	Single	19	7.0%
	Divorce	2	0.7%
Number of children	0	36	13.8%
	1	64	24.6%
	2	89	34.2%
	3	41	15.8%
	4	23	8.8%
	5	7	2.7%
	>=6	11	4.1%
Do you work night shifts?	Yes	228	84.1%
	No	43	15.9%
Do you have any medical illness that prevents you from falling asleep or continuing?	Yes	56	20.7%
	No	215	79.3%
If yes, choose one	HTN	10	17.9%
	DM	3	5.4%
	Pain	26	46.4%
	Other	14	25.0%
	I don't have any medical illness	3	5.4%
Have you taken any sleeping pills for the last 3 months?	Yes	18	6.6%
	No	253	93.4%
If yes, how frequently per week?	Zero‎/week	38	15.0%
	Once/week	0	0.0%
	2 times‎/week	1	0.4%
	3 times/week	0	0.0%
	Equal to or more than 4 times‎/week	2	0.8%
	I haven't taken any sleeping pills for the last 3 months	212	83.8%

**Figure 1:**
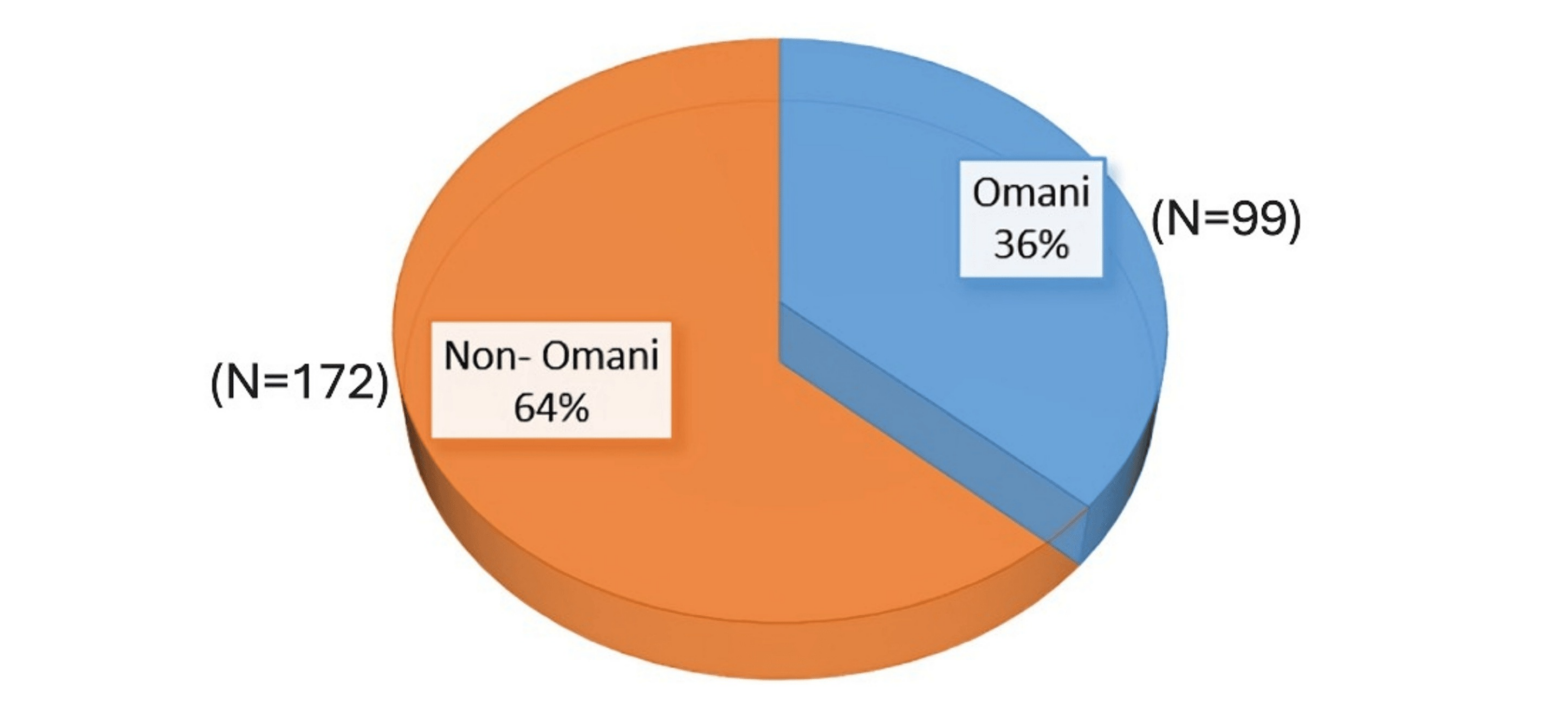
Nurses nationality distribution.

This can be seen as well in Figure 1.

**Figure 2:**
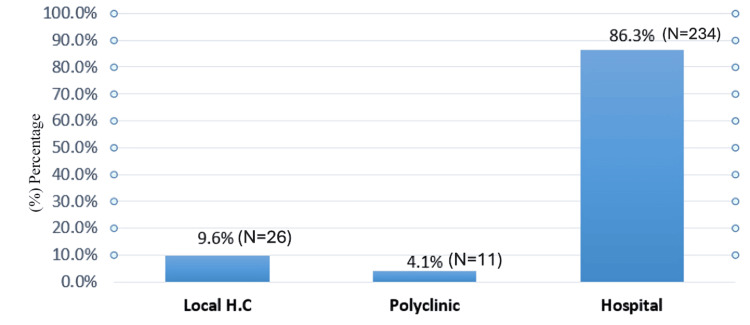
Workplace distribution. Local H.C: local health centre.

Most participants worked in hospital settings (N=234, 86.3%), followed by local health centres (N=26, 9.6%) and polyclinics (N=11, 4.1%) (Figure 2).

**Figure 3:**
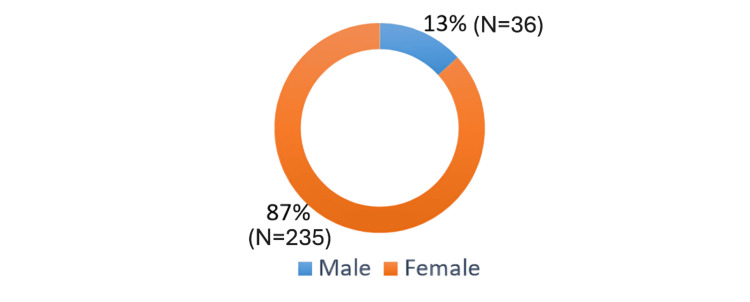
Gender distribution.

A large proportion of respondents were female (N=235, 86.7%), while 13.3% (N=36) were male (Figure 3).

**Figure 4:**
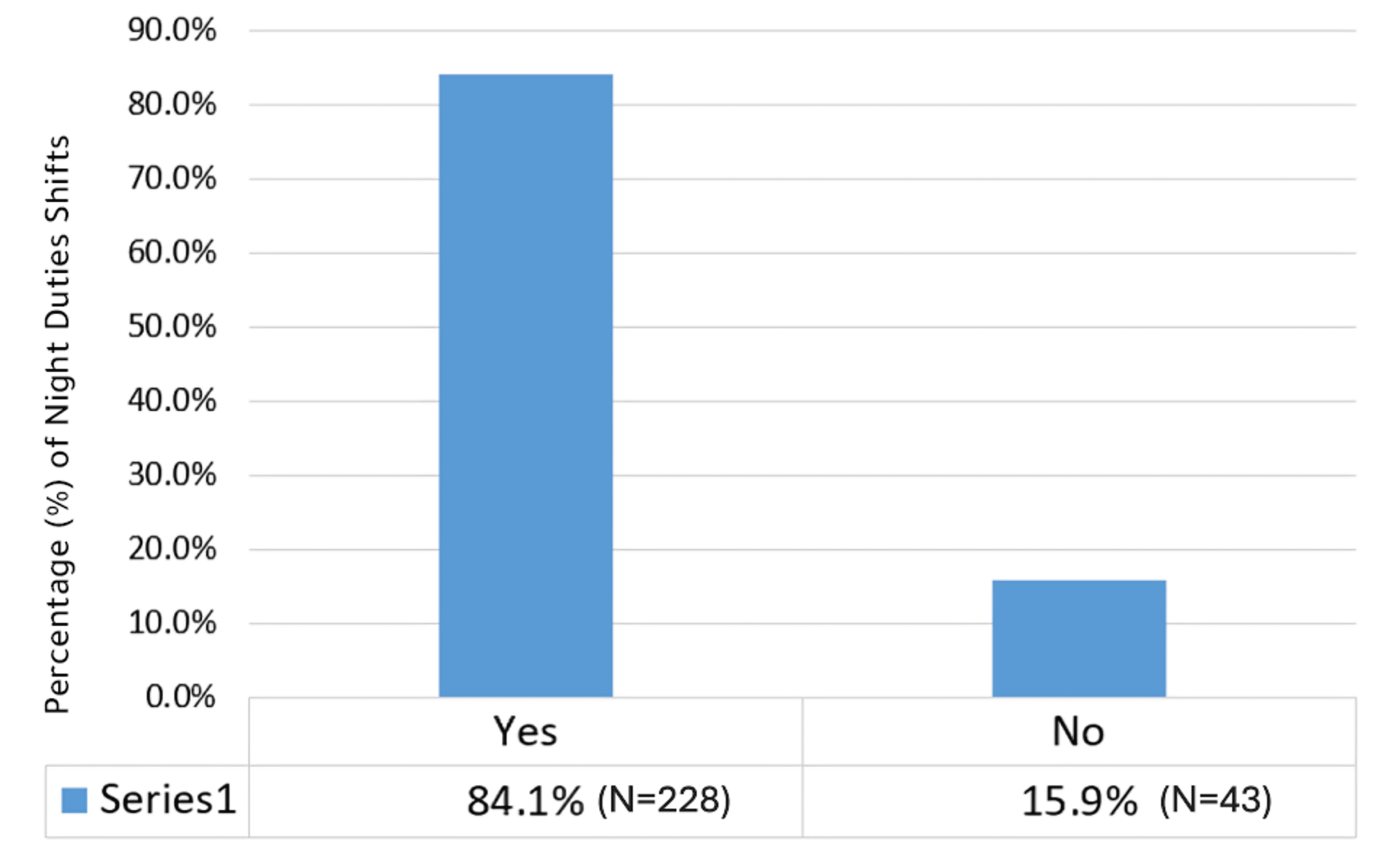
Night shifts duties distribution.

Regarding experience, most nurses had worked 15-20 years (N=86, 31.7%), and the largest age group was 30-39 years (N=163, 60.1%). Over half of the participants (N=155, 57.2%) had been working in their current department for 1-5 years, and a majority were married (N=250, 92.3%). About 61.1% (N=171) of nurses had more than one child. The vast majority (N=228, 84.1%) reported working night shifts (Figure 4), and 20.7% (N=57) had a medical illness affecting their sleep. Among those, pain (46.4%) was the most common condition. Only 6.6% (N=18) reported using sleeping pills during the last three months, and most of them used these less than once per week.

Prevalence and severity of insomnia among nurses in Al-Dakhaliah Governorate

Table 2 summarizes the prevalence and severity of insomnia. ISI total scores showed: 42.4% (N=115) had no clinically significant insomnia (ISI: 0-7), 33.6% (N=91) had subthreshold insomnia (ISI: 8-14), 21.4% (N=58) had moderate insomnia (ISI: 15-21), and 2.6% (N=7) had severe insomnia (ISI: 22-28).

**Table 2:** Insomnia Severity Index category distribution.

ISI Category	Frequency	Percentage
No clinically significant insomnia (0-7)	115	42.4 %
Subthreshold insomnia (8-14)	91	33.6%
Moderate clinical insomnia (15-21)	58	21.4%
Severe clinical insomnia (22-28)	7	2.6%
Total	271	100.0%

**Figure 5:**
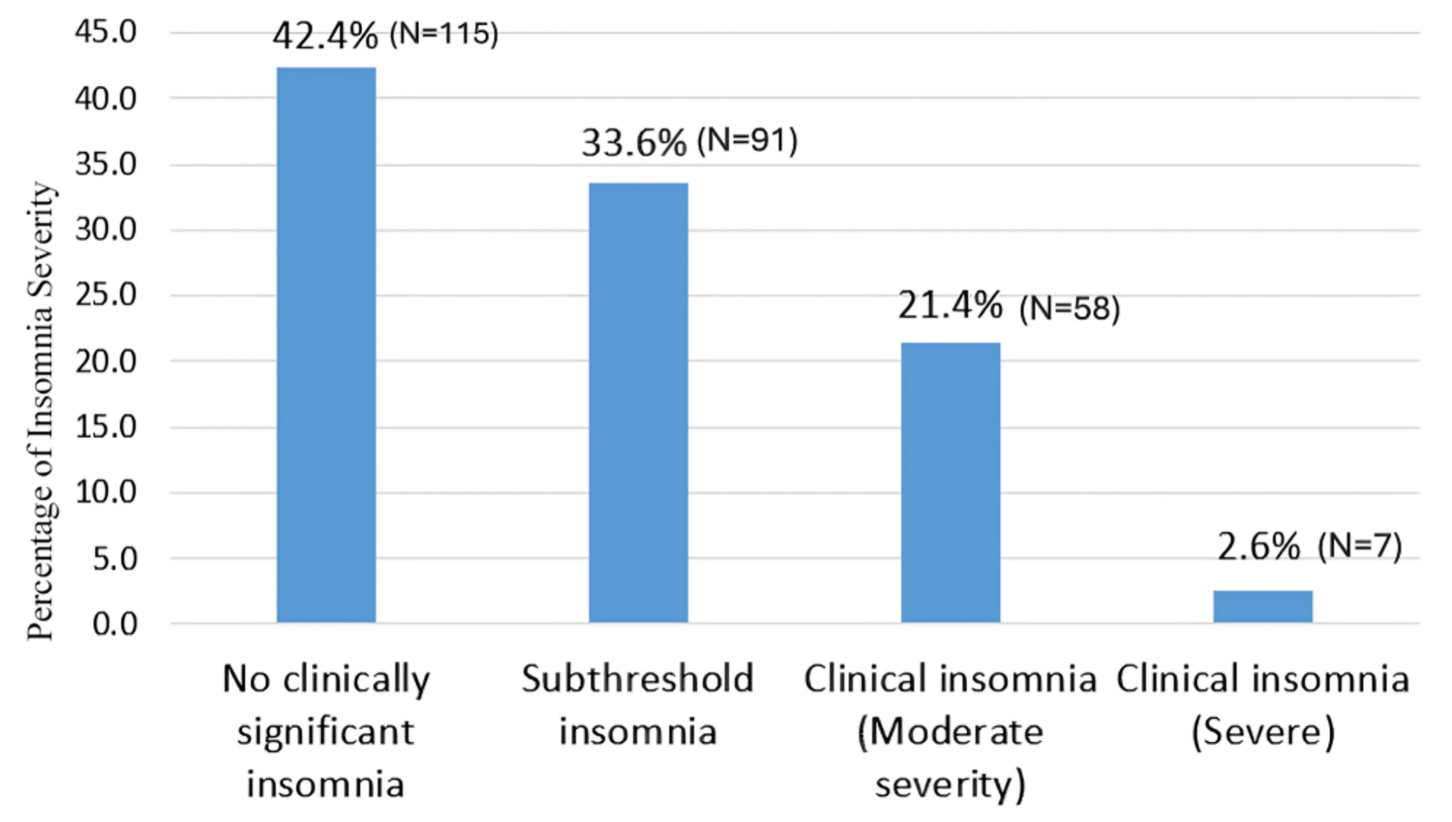
Percentage of prevalence and severity of insomnia (Insomnia Severity Index).

Combined clinically significant insomnia (moderate + severe) was 24.0% (N=65) (Figure 5). This indicates that approximately one-quarter of the nurses had clinically significant insomnia and one-third had subthreshold insomnia, reflecting a substantial burden of sleep disturbance in this population.

Table 3 presents the means and standard deviations for each item of the ISI. Participants reported the highest severity on the item Satisfaction with recent sleep patterns (M=1.76, SD=1.19), suggesting lower satisfaction with their recent sleep quality.

**Table 3:** Mean and SD of Insomnia Severity Index (ISI) items score. Range for all the variables is 0-4.

Item	Mean	SD
Difficulty falling asleep (initial insomnia)	1.17	1.08
Difficulty maintaining sleep (sleep maintenance difficulty)	1.20	1.00
Early morning awakening (terminal insomnia)	1.15	1.05
Satisfaction with recent sleep patterns	1.76	1.19
Sleep disturbance interferes with daily functioning	1.48	1.09
Noticeability of sleep quality impairment to others	1.30	1.04
Concern/distress about recent sleep problems	1.40	1.17
Total ISI	9.46	6.30

**Figure 6:**
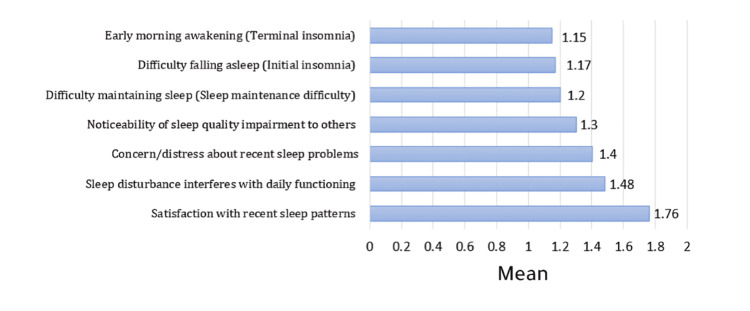
Means of Insomnia Severity Index items (0-4).

The lowest severity was observed on early morning awakening (terminal insomnia) (M=1.15, SD=1.05), indicating fewer difficulties with waking up too early (Figure 6). The mean total ISI score was 9.46 (SD=6.30), which falls within the subthreshold insomnia range (8-14) according to standard ISI scoring guidelines. Overall, these findings suggest that participants experienced mild sleep difficulties, with dissatisfaction regarding sleep patterns being the most prominent concern. 

The findings in Table 4 indicate that most demographic and work-related characteristics of nurses were not significantly associated with insomnia severity. Variables such as workplace setting, gender, age, years of nursing experience, years working in the current department, marital status, number of children, and night shift duties did not show any meaningful differences in ISI scores (p> 0.05 for all). In contrast, three variables demonstrated statistically significant relationships with insomnia severity. Omani nurses reported significantly higher insomnia scores than non-Omani nurses (p< 0.001), suggesting potential cultural, social, or work-related stress differences between groups. Nurses with a medical illness had markedly higher insomnia levels compared to those without medical conditions (p< 0.001), indicating that underlying health problems may intensify sleep disturbances. Additionally, those who reported using sleeping pills during the past three months had the highest ISI scores among all groups (p< 0.001), which may reflect more severe or persistent sleep problems that necessitate medication. Overall, while most background characteristics did not contribute to variations in insomnia severity, nationality, presence of medical illness, and sleeping pill usage emerged as key factors associated with significantly higher insomnia symptoms among nurses. 

**Table 4:** Mean Insomnia Severity index scores according to background characteristics and profile of medication usage (n=271).

Variable	Category	N	(%)	Mean	SD	t/F-Test	p-value	Interpretation
Nationality	Omani	99	36.5	11.8	5.4	t=4.84	<0.001	Statistically significant
	Non-Omani	172	63.5	8.11	6.4			
Workplace	Local H.C	26	9.6	10.38	5.32	F=1.59	0.227	Not statistically significant
	Polyclinic	11	4.1	12.36	6.53			
	Hospital	234	86.3	9.22	6.37			
Gender	Male	36	13.3	8.69	5.98	t=-0.78	0.434	Not statistically significant
	Female	235	86.7	9.58	6.35			
How many years worked as a nurse?	1-4	28	10.3	8.75	6.1	F=0.531	0.713	Not statistically significant
	5-9	52	19.2	9.94	6.38			
	10-14	77	28.4	9.74	6.85			
	15-20	86	31.7	8.87	5.82			
	21-30	28	10.3	10.32	6.41			
Age group	20-29	27	10.0	10.59	5.57	F=0.78	0.52	Not statistically significant
	30-39	163	60.1	9.01	6.28			
	40-49	76	28.0	10	6.64			
	50-59	5	1.8	10	5.7			
How many years worked as a nurse at the current department?	1-5	155	57.2	8.97	6.26	F=1.09	0.402	Not statistically significant
	6-10	43	15.9	9.23	6.5			
	11-15	45	16.6	10.62	5.96			
	16-20	16	5.9	12.38	6.43			
	21-25	9	3.3	8.11	7.36			
	26-30	3	1.1	9.33	4.93			
Marital status	Married	250	92.3	9.52	6.29	F=0.11	0.898	Not statistically significant
	Single	19	7.0	8.74	6.23			
	Divorce	2	0.7	9	12.73			
Number of children	0	36	13.3	9	6.12	F=2.22	0.057	Not statistically significant
	1	64	23.6	8.45	5.4			
	2	89	32.8	8.98	6.82			
	3	41	15.1	10.24	6.59			
	4	23	8.5	11.09	6.22			
	5	7	2.6	13.71	3.99			
	>=6	11	4.1	11.73	6.63			
Work night shifts	No	43	15.9	11	5.76	t=1.7	0.091	Not statistically significant
	Yes	228	84.1	9.18	6.37			
Do you have any medical illnesses?	No	215	79.3	8.31	6.02	t=-6.32	<0.001	Statistically significant
	Yes	56	20.7	13.9	5.35			
Have you taken any sleeping pills for the last 3 months?	No	253	93.4	9.08	6.26	t=-3.84	<0.001	Statistically significant
	Yes	18	6.6	14.8	4.18			

Table 5 presents the summary results of a multiple linear regression analysis conducted to determine the factors associated with insomnia severity among nurses, as measured by the ISI score. All variables were entered simultaneously to adjust for potential confounding effects. The overall model explained 25.3% of the variance in insomnia severity (R²=0.253), with an adjusted R² of 0.152, indicating a moderate level of explanatory power. After controlling for all other demographic and work-related variables, only two variables demonstrated statistically significant associations with insomnia severity: nationality and medical illness.

**Table 5:** Multiple linear regression model summary.

R	R²	Adjusted R²	Std. error estimate
0.5030	0.2530	0.1521	5.8179

The findings in Table 6 indicate that after adjusting for all predictors, nationality and medical illness remained significant independent predictors of insomnia severity. Non-Omani nurses had lower insomnia scores than Omani nurses, while nurses with medical illnesses showed markedly higher levels of insomnia symptoms. No other demographic or work-related factors demonstrated significant associations in the final model. Non-Omani nurses had significantly lower ISI scores compared with Omani nurses (B=−2.7, p=0.028; 95% CI: −5.2 to −0.3). This suggests that being Omani is associated with greater insomnia symptoms. Nurses reporting a medical illness had significantly higher ISI scores than those without (B=4.32, p< 0.001; 95% CI: 2.27 to 6.37). This was the strongest predictor in the model (β=0.28).

**Table 6:** Predictors of nurses’ insomnia (multiple linear regression, n=271). Local HC: local health centre.

Parameter		Non standard coef beta	Standard coef beta	Std. error	t	p-value	95% CI	
							lower bound	upper bound
Constant		6.89	-	5.35	1.29	0.2	-3.7	17.4
Nationality (reference: Omani)								
Non-Omani		-2.7	-0.2	1.23	-2.2	0.03	-5.2	-0.3
Workplace (reference: polyclinic)								
Hospital		-0.9	-0.1	2.11	-0.4	0.66	-5.1	3.22
Local HC		-1.6	-0.1	2.29	-0.7	0.48	-6.1	2.89
Gender (reference: male)								
Gender female		2.22	0.12	1.28	1.74	0.08	-0.3	4.75
Years worked as a nurse (reference: 1-4)								
5-9 years		3.37	0.21	1.74	1.94	0.05	-0.1	6.8
10-14 years		2.9	0.21	1.82	1.59	0.11	-0.7	6.5
15-20 years		-0.1	0	1.96	-0.1	0.96	-4	3.75
21-30 years		0.62	0.03	2.61	0.24	0.81	-4.5	5.77
Age group (reference: 20-29)								
30-39 years		-2.5	-0.2	1.79	-1.4	0.16	-6.1	1
40-49 years		-1.1	-0.1	2.13	-0.5	0.61	-5.3	3.1
50-59 years		0.46	0.01	4.15	0.11	0.91	-7.7	8.64
Years in at current department (reference: 1-5)								
6-10 years		0.42	0.02	1.1	0.38	0.7	-1.7	2.59
11-15 years		1.35	0.08	1.16	1.16	0.25	-0.9	3.64
16-20 years		1.32	0.05	1.91	0.69	0.49	-2.4	5.08
21-25 years		-1.8	0	2.57	-0.7	0.49	-6.9	3.27
26-30 years		-4.8	-0.1	4.69	-1	0.31	-14	4.47
Marital status (reference: divorced)								
Married		-0.5	0	4.38	-0.1	0.91	-9.1	8.15
Single		-1.7	-0.1	4.9	-0.4	0.72	-11	7.93
Number of children (reference: 0)								
1		-1.2	-0.1	1.63	-0.7	0.48	-4.4	2.04
2		0.72	0.05	1.58	0.45	0.65	-2.4	3.83
3		0.47	0.03	1.82	0.26	0.8	-3.1	4.07
4		0.53	0.02	2.26	0.23	0.82	-3.9	4.99
5		3.25	0.08	3.03	1.07	0.29	-2.7	9.22
>=6		1.96	0.06	3.07	0.64	0.52	-4.1	8
Do you work night shifts (reference: no)								
Yes		1.55	0.09	1.5	1.03	0.3	-1.4	4.49
Medial illness (reference: no)								
Yes		4.32	0.28	1.04	4.15	< 0.001>	2.27	6.37
Sleeping pills (reference: no)								
Yes		2.9	0.11	1.69	1.72	0.09	-0.4	6.22
How frequent per week (reference: 0)								
2 times/week		2.62	0.03	6.04	0.43	0.67	-9.3	14.5
>=4 times/week		-0.5	0	4.59	-0.1	0.91	-9.6	8.52
I don't take		1.68	0.1	1.1	1.53	0.13	-0.5	3.85

## Discussion

In the present study, medical illness and nationality emerged as the only significant predictors of insomnia severity among nurses after controlling for demographic and occupational variables. Nurses who reported having a medical illness experienced significantly higher insomnia scores [5]. This finding is consistent with previous studies indicating that chronic health conditions can impair sleep quality through physical discomfort, pain, stress, and psychological burden [6,8,9,10]. Nurses with existing health issues may also be more vulnerable to work-related strain, which can further exacerbate sleep disturbances [6,11]. Early identification and supportive workplace accommodations may therefore be critical to reduce sleep problems among this group [5,6,12,13]. 

Interestingly, non-Omani nurses demonstrated significantly lower insomnia severity compared with Omani nurses. This result contrasts with expectations, as expatriate nurses may commonly encounter stressors such as workload adaptation, homesickness, cultural challenges, and job insecurity [14,15]. One possible explanation is that non-Omani nurses may adopt more adaptive coping strategies or have stronger family or peer support systems. Alternatively, Omani nurses may be facing increased national responsibilities, societal expectations, or dual-role pressures within the home and workplace, leading to more sleep disruptions. Further qualitative research is recommended to explore these sociocultural factors [16,17]. 

Other demographic and work-related characteristics, such as age, gender, workplace setting, night shift status, and years of experience, were not significantly associated with insomnia in the final adjusted model. This suggests that insomnia among nurses may be more strongly driven by personal health conditions and psychosocial influences rather than routine workforce characteristics. It also indicates that common occupational assumptions, such as night shift work being the primary cause of poor sleep, may not fully capture the complexity of insomnia in this population [3,15]. The modest explanatory power of the model (adjusted R²=0.152) further suggests that additional relevant variables were not captured, such as occupational stress, burnout, staffing level, workload intensity, and mental health factors (e.g., anxiety and depression), which are known contributors to sleep disorders in nursing staff [6,12]. Future research should include a broader range of psychosocial predictors to better understand the multifactorial nature of insomnia among nurses. 

Overall, the findings highlight the importance of addressing medical conditions and supporting Omani nurses who appear more susceptible to insomnia. Tailored sleep health interventions and workplace wellbeing programs may reduce the burden of sleep disturbances and enhance nursing performance and patient safety [1,2,4,18,19]. 

A comprehensive, multi-level strategy to guide insomnia treatment among nurses

Health Management

Routine screening and optimal management of chronic medical illnesses are essential to reduce physiological drivers of sleep disruption.

Stress Reduction

Evidence-based interventions such as Mindfulness-Based Stress Reduction (MBSR) and Cognitive Behavioral Therapy for Insomnia (CBT-I) should be implemented to address psychological stressors and maladaptive sleep behaviors.

Shift Scheduling

Forward-rotating shift systems, protected rest breaks, and access to designated rest areas can minimize circadian misalignment, a core mechanism in shift-work related insomnia.

Mental Health Support

Formal fatigue management and burnout prevention programs are critical for lowering insomnia severity and improving emotional resilience in high-demand work settings.

Medication Use

Pharmacotherapy should be considered adjunctive and time-limited. The gold-standard approach involves CBT-I combined with gradual tapering of hypnotics to reduce dependence and avoid rebound insomnia.

Cultural Sensitivity

Interventions should be culturally adapted, family-inclusive, and context-appropriate for Omani nurses to improve acceptability, engagement, and sustainability.

Recommendations

Comprehensive health screening and appropriate workplace accommodations should be provided for nurses living with chronic illnesses to support their well-being and job performance. Moreover, access to CBT-I and mindfulness-based programs may help reduce reliance on hypnotic medications while promoting healthier sleep patterns and implementing evidence-based rostering practices that ensure adequate recovery periods, along with education on sleep hygiene, can further optimize nurses’ sleep quality and overall functioning. Additionally, culturally tailored well-being initiatives, including family-inclusive supports, are particularly important for Omani nurses. Finally, future prospective studies should explore predictors such as burnout, workload, and mental health to better understand factors influencing sleep disturbances among nurses.

Strengths and limitations

This study is one of the very few investigations focusing specifically on sleep disturbance and insomnia among nurses working within the Al-Dakhiliyah Governorate. The use of a validated insomnia screening tool (Insomnia Severity Index) strengthens the reliability of the findings, and the inclusion of nurses from different levels of healthcare institutions (hospitals, polyclinics, and local health centres) increases the contextual relevance and representativeness of the results within the governorate. Additionally, the analysis applied both bivariate and multivariable regression techniques, allowing identification of independent predictors beyond basic demographic differences.

However, this study has limitations that should be acknowledged. First, data were collected using convenience sampling, which limits the generalizability of findings and may introduce response bias, particularly if nurses experiencing sleep disturbance were more motivated to participate. Second, the cross-sectional nature of the study prevents the determination of causality; thus, associations do not imply directionality. Third, data collection relied on self-reported measures, which may be affected by recall bias or social desirability bias. Finally, important psychosocial factors known to influence sleep, such as workload intensity, burnout, staffing adequacy, and mental health variables (anxiety, depression), were not measured in this study and may partially explain the unexplained variance in ISI scores. Future research should incorporate longitudinal designs, structured mental health screening, and objective sleep measures (e.g., actigraphy) to better understand the causal mechanisms underlying insomnia among nurses in Oman.

## Conclusions

Insomnia was found to be a modest prevalent condition among nurses working in Al-Dakhiliyah Governorate, with many experiencing clinically significant insomnia and reporting subthreshold symptoms. Although most demographic and occupational variables were not associated with insomnia severity, both medical illness and nationality emerged as independent predictors. Nurses with chronic health conditions demonstrated markedly higher insomnia scores, underscoring the role of physical health in sleep disturbance. Furthermore, Omani nurses exhibited significantly higher insomnia severity than non-Omani nurses, suggesting that sociocultural and family-role pressures may contribute to sleep impairment within the local nursing workforce.

These findings highlight the need for targeted sleep health strategies within the Omani nursing system. Routine screening for chronic illness, incorporation of structured sleep-focused wellbeing initiatives, and access to evidence-based non-pharmacological interventions such as CBT-I may reduce insomnia burden and enhance occupational functioning. Future research should integrate longitudinal designs and include psychosocial, mental health, burnout, and workload factors to strengthen causal inference and guide intervention development. Improving sleep health in nurses is a strategic priority to protect workforce wellbeing and maintain the quality and safety of patient care.

## Appendices

Appendix A

**Table 7:** Questionnaire used in the study.

Components	Items						
Nationality	Omani	Non-Omani	-	-	-	-	-
Workplace	Local H-C	Polyclinic	Hospital	-	-	-	-
Gender	Female	Male	-	-	-	-	-
How many years worked as a nurse?	1-4	5-9	10-14	15-20	21-30	-	-
Age	22-29	30-39	40-49	50-59	>60	-	-
How many years worked as a nurse at the current department?	1-5	6-10	11-15	16-20	21-25	26-30	-
Marital status	Married	Single	Divorced	-	-	-	-
Number of children	0	1	2	3	4	5	>6
Do you work night shifts?	Yes	No	-	-	-	-	-
Do you have any medical illness that prevents you from falling asleep or continuing to sleep:	Yes	No	-	-	-	-	-
If yes, choose one	HTN	DM	pain	other	I don't have any medical illness.	-	-
Have you taken any sleeping pills for the last 3 months?	Yes	No	-	-	-	-	-
If yes, how frequently per week?	Zero/week	Once a week	2 times a week	3 times a week.	Equal to or more than 4 times a week.	I haven't taken any sleeping pills for the last 3 months.	
Please rate the current (i.e., last week) severity of your insomnia problem(s).	0 (<30 min/none)	1 (30-45 min/mild)	2 (45-90 min/moderate)	3 (90-120 min/severe)	4 (>120 min/very)	-	-
Difficulty staying asleep	0 (None)	1 (Mild)	2 (Moderate)	3 (Severe)	4 (Very)	-	-
Problem waking up too early	1 (None)	2 (Mild)	3 (Moderate)	4 (Severe)	5 (Very)	-	-
How satisfied/dissatisfied are you with your current sleep pattern?	0 (Very satisfied).	1	2	3	4 (Very dissatisfied).	-	-
To what extent do you consider your sleep problem to interfere with your daily functioning (e.g. daytime fatigue, ability to function at work/daily chores, concentration, memory, mood, etc.)?	0 (Not at all interfering)	1 (A little)	2 (Somewhat)	3 (Much)	4 (Very much interfering)	-	-
How noticeable to others do you think your sleeping problem is in terms of impairing the quality of your life?	0 (Not at all noticeable)	1 (Barely)	2 (Somewhat)	3 (Much)	4 (Very much noticeable)	-	-
How worried/distressed are you about your current sleep problem?	0 (Not at all)	1 (A little)	2(Somewhat)	3 (Much)	4 (Very much)	-	-
